# Propolin G-Suppressed Epithelial-to-Mesenchymal Transition in Triple-Negative Breast Cancer Cells via Glycogen Synthase Kinase 3β-Mediated Snail and HDAC6-Regulated Vimentin Degradation

**DOI:** 10.3390/ijms23031672

**Published:** 2022-01-31

**Authors:** Jih-Tung Pai, Xing-Han Chen, Yann-Lii Leu, Meng-Shih Weng

**Affiliations:** 1 Division of Hematology and Oncology, Tao-Yuan General Hospital, Ministry of Health and Welfare, Taoyuan City 33004, Taiwan; jihtungpai@gmail.com; 2 Department of Nutritional Science, Fu Jen Catholic University, New Taipei City 24205, Taiwan; bootys840812@gmail.com; 3 Graduate Institute of Natural Products, College of Medicine, Chang Gung University, Taoyuan City 33302, Taiwan; ylleu@mail.cgu.edu.tw; 4 Tissue Bank, Chang Gung Memorial Hospital, Linkou, Taoyuan City 33342, Taiwan

**Keywords:** triple-negative breast cancer (TNBC), epithelial-to-mesenchymal transition (EMT), propolin G, glycogen synthase kinase 3β, (GSK-3β), histone deacetylase 6 (HDAC6)

## Abstract

Triple-negative breast cancer (TNBC) is a highly aggressive breast cancer with a poor prognosis. The incidence and mortality rate of TNBC are frequently found in younger women. Due to the absence of a good therapeutic strategy, effective remedies for inhibiting TNBC have been developed for improving the cure rate. Epithelial-to-mesenchymal transition (EMT) is a critical mechanism to regulate cancer cell motility and invasion. Furthermore, ectopic expression of EMT molecules correlates with the metastasis and poor prognosis of TNBC. Targeting EMT might be a strategy for the therapy and prevention of TNBC. Propolin G, an active c-prenylflavanone in Taiwanese propolis, has been shown to possess anti-cancer activity in many cancers. However, the anti-metastasis activity of propolin G on TNBC is still unclear. The present study showed that the migration and invasion activities of TNBC cells was suppressed by propolin G. Down-regulated expression of Snail and vimentin and up-regulated expression of E-cadherin were dose- and time-dependently observed in propolin G-treated MDA-MB-231 cells. Propolin G inhibited Snail and vimentin expressions via the signaling pathways associated with post-translational modification. The activation of glycogen synthase kinase 3β (GSK-3β) by propolin G resulted in increasing GSK-3β interaction with Snail. Consequently, the nuclear localization and stability of Snail was disrupted resulting in promoting the degradation. Propolin G-inhibited Snail expression and the activities of migration and invasion were reversed by GSK-3β inhibitor pretreatment. Meanwhile, the outcomes also revealed that histone deacetylase 6 (HDAC6) activity was dose-dependently suppressed by propolin G. Correspondently, the amounts of acetyl-α-tubulin, a down-stream substrate of HDAC6, were increased. Dissociation of HDAC6/Hsp90 with vimentin leading to increased vimentin acetylation and degradation was perceived in the cells with the addition of propolin G. Moreover, up-regulated expression of acetyl-α-tubulin by propolin G was attenuated by HDAC6 overexpression. On the contrary, down-regulated expression of vimentin, cell migration and invasion by propolin G were overturned by HDAC6 overexpression. Conclusively, restraint cell migration and invasion of TNBC by propolin G were activated by the expression of GSK-3β-suppressed Snail and the interruption of HDAC6-mediated vimentin protein stability. Aiming at EMT, propolin G might be a potential candidate for TNBC therapy.

## 1. Introduction

Distant metastasis of cancer is an important mechanism causing cancer death. Cancer metastasis is a complex mechanism that involves cancer cells migrating and invading to the surrounding tissue, intravasating into the vasculature, and extravasating to distant tissue [1]. Disruption of cell junction and cell-to-extracellular matrix (ECM) connection by systemic mechanisms and proteolytic enzymes are critical steps for triggering cell metastasis. Epithelial-to-mesenchymal transition (EMT) is a process to promote cells to lose epithelial cell polarity and characteristics followed by attaining mesenchymal cell properties, which break out of the cell junction and initiate cell migration and invasion [2]. During the EMT process, the expression of E-cadherin, an epithelial cell molecule, is obviously decreased. Conversely, N-cadherin and vimentin of the mesenchymal cell markers as well as EMT-regulated transcriptional factors, the Snail family, are significantly up-regulated [3]. Reversing or restraining EMT molecular profile expressions might be a strategy for suppressing cancer cell migration and invasion ensuing ceasing metastasis progression.

Breast cancer is the most common cancer of females and ranks in the first place to cause cancer death of women in Western countries [4,5]. Breast cancer is categorized into luminal A, luminal B, the human epidermal growth factor receptor 2 (HER-2)-overexpressed, and basal-like subtypes [6]. Triple-negative breast cancer (TNBC) is distinct but similar to the basal-like subtype of breast cancer and possesses a high aggressive property and poor prognosis. The deficient expressions of hormone receptors (estrogen receptor (ER) and progesterone receptor (PR)) expressions and HER-2 gene amplification are the critical features of TNBC. Although TNBC only accounts for 10–20% of breast cancer, the mortality rate is higher than other subtypes of breast cancer. Furthermore, the incidence of TNBC is more frequent and more prevalent in younger women than in the elderly females [7,8]. The younger population is in urgent need of rescue from this serious threat of TNBC. Unfortunately, conventional targets and endocrine therapies are invalid for the treatment of TNBC currently. Developing a suitable strategy for TNBC treatment to promote the patient’s survival rate is essential. Numerous studies indicate that the development of metastasis is the decisive reason that the overall survival rate of TNBC is so low [9,10,11]. Thus, understanding the mechanisms of metastasis might provide insights for improving the survival of TNBC patients.

Protein modifications by phosphorylation, acetylation, and/or ubiquitination are the key mechanisms to regulate cell malignancy [12]. The stabilities and functions of numerous EMT molecules are controlled by post-translational modifications. The stabilities of Snail and β-catenin proteins and the nuclear localization regulated by phosphorylation modification through glycogen synthase kinase-3β (GSK-3β)-mediated cell migration and invasion are designated [13,14,15]. The phosphorylations via protein kinase C (PKC) and Rho family GTPase-up-regulated vimentin are involved in increasing cell migration capability [16]. Furthermore, phosphorylation of vimentin on Ser39 by Akt is also observed in invasive cancer cells and metastatic tumors [17]. In addition to phosphorylation, acetylation modification is another mechanism that governs protein stability. The deacetylations of histone and some non-histone proteins on lysine residue are catalyzed by histone deacetylases (HDACs). HDACs are classified as class I (HDAC1–3 and 8), class IIa (HDAC4, 5, 7, and 9), class IIb (HDAC6 and 10), class III (SIRT1–7), and class IV (HDAC11) [18,19]. HDAC6 is mainly localized in cytoplasm and catalyzes deacetylation of non-histone protein, such as p53, epidermal growth factor receptor (EGFR), matrix metalloproteinase 9 (MMP9), and cyclin-dependent kinase [20,21,22,23]. Suppressing MMP9-mediated lung cancer cell migration and invasion through the repression of HDAC6 activity has been demonstrated [22]. The regulations of breast cancer cell invadopodia formation and invasion are through the HDAC6-dependent signaling pathway [24]. Additionally, the HDAC6-regulated collapse of the vimentin intermediate filament network is involved in cellular stiffness and cell invasion [25]. Overexpression of non-acetylated vimentin attenuates the migration activity of hepatocellular carcinoma cells [26]. Thus, targeting protein modifications might be a suitable strategy for EMT suppression leading to restrain cancer cell migration and invasion.

Propolis is a resinous material collected by bees and has been used as a folk medicine for at least a thousand years. The constituents of propolis are a mixture of plant exudates and some metabolites of bees [27,28]. The categories of biological components of propolis depend upon the species of plant sources. According to the geographic distribution, propolis is divided into six categories around the world [27]. The most abundant contents are polyphenols, terpenoids, and prenylated derivates of *p*-coumaric acids [29,30]. The specific active compound category, c-prenylflavanones, was discovered and identified in Taiwanese and Okinawan propolis [31,32,33]. So far, eight prenylflavanones have been purified and identified in Taiwanese propolis and named propolin A to H. Propolins have been revealed to possess anti-bacterial, anti-oxidant, anti-inflammation, and anti-cancer activities [33,34,35]. Inhibition of cancer cell proliferation by propolins through cell cycle arrest and the induction of intrinsic apoptosis mechanisms has been demonstrated in many cancer cells [36,37,38,39,40]. Moreover, propolin C-disrupted EGFR-regulated EMT involves in the suppression of lung cancer cell migration and invasion [41]. Although EMT has been implied as a target of propolins in migration and invasion inhibition, the molecular mechanisms are still a mystery, especially in TNBC. The present study appraised the role of EMT protein modifications by propolin G, a natural HDAC6 inhibitor in Taiwanese propolis, in anti-migration and anti-invasion activity of TNBC cells. The results discovered that the repression of cell migration and invasion activity via propolin G was stimulated by the protein degradations of Snail and vimentin through disrupting the protein modification system in TNBC cells. Propolin G might be an appropriate candidate for TNBC therapy and/or prevention.

## 2. Results

### 2.1. The Migration and Invasion Abilities of TNBC Cell Lines

**Figure 1:**
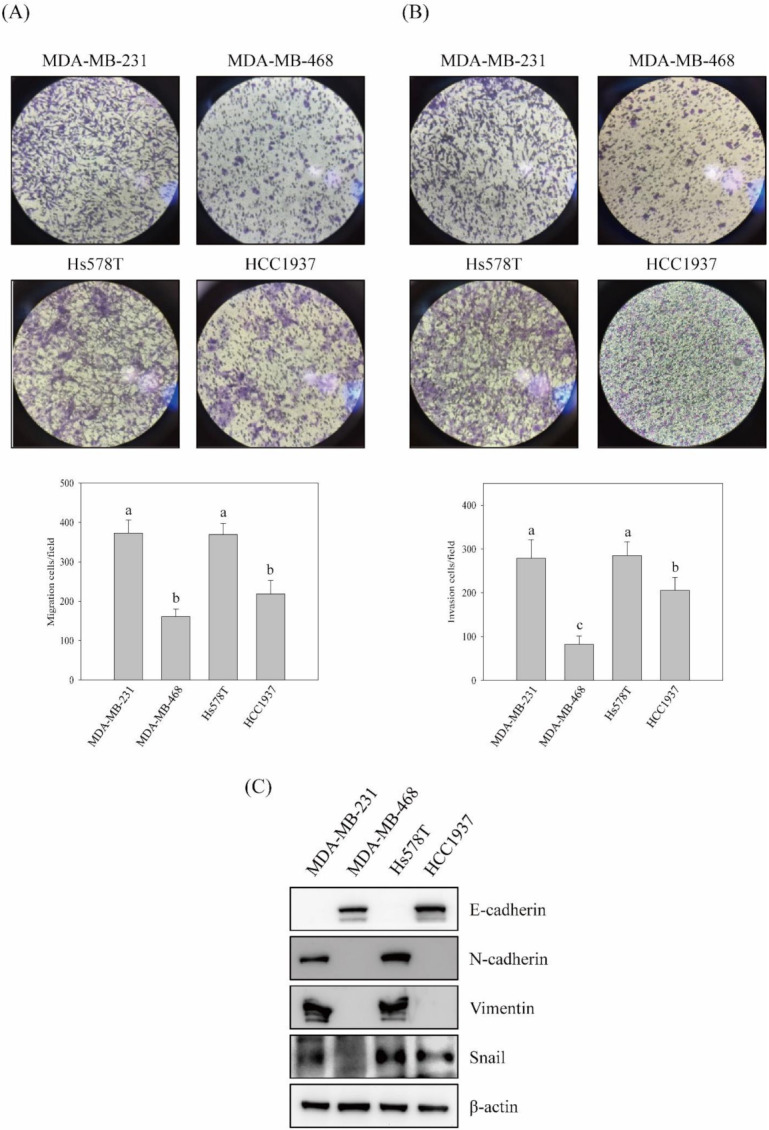
The migration and invasion activities in vitro and the expression of EMT-associated molecules in triple-negative breast cancer cell lines. Triple-negative breast cancer (TNBC) cell lines MDA-MB-231, MDA-MB-468, Hs578T, and HCC1937 were cultured and in vitro activities of (**A**) migration and (**B**) invasion were performed as described in Materials and Methods. (**C**) The protein expressions of EMT-associated molecules in TNBC cell lines were inspected by Western blotting analyses. Data are presented for at least triplicate experiments, and results are represented as the mean ± S.D. Different lowercase letters represent significant differences between the two groups when *p* < 0.05.

To understand the metastatic ability of TNBC cells, the in vitro migration and invasion activities and the expressions of EMT molecules of four TNBC cell lines were evaluated. As shown in Figure 1, the high migration and invasion activities were detected in MDA-MB-231 and Hs578T cells (Figure 1A,B). Furthermore, the expression of E-cadherin was clearly perceived in the low migration and invasion ability cells, MDA-MB-468 and HCC1937. On the contrary, the expressions of N-cadherin, vimentin, and Snail were obviously observed in MDA-MB-231 and Hs578T cells (Figure 1C). However, the expression of Snail was also significantly displayed in HCC1937 cells.

### 2.2. Migration and Invasion Activities Suppressed by Propolin G in TNBC Cells

**Figure 2:**
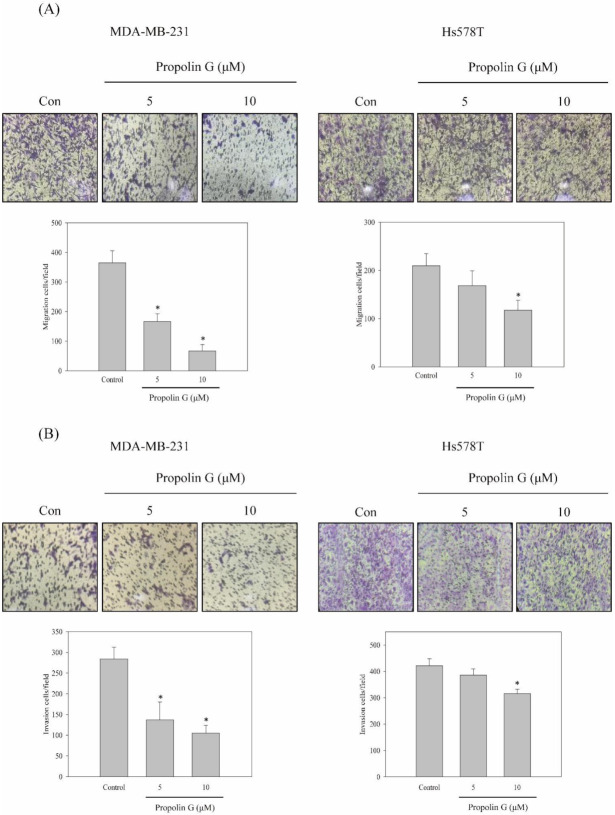

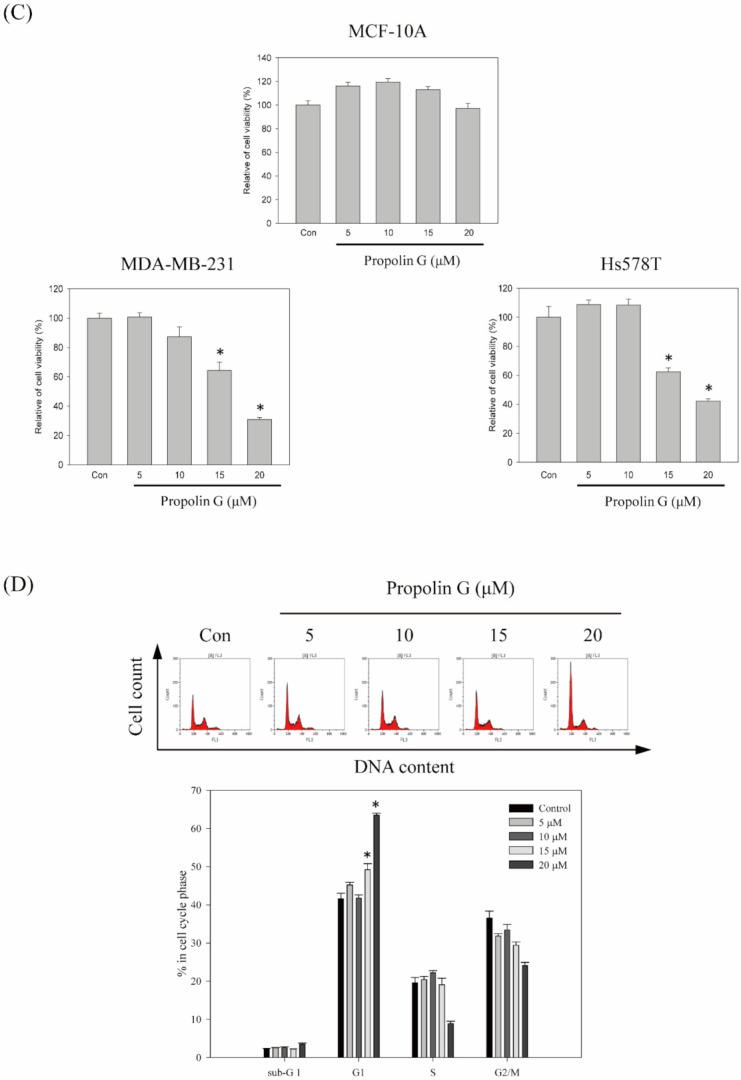
Suppression of the in vitro cell migration and invasion activities of TNBC cell lines by propolin G. MDA-MB-231 and Hs578T cells were cultured and treated with indicated concentrations of propolin G (0, 5, and 10 μM) for 24 h, and the in vitro (**A**) migration and (**B**) invasion analyses were determined as described in Materials and Methods. (**C**) MDA-MB-231, Hs578T, and MCF10A cells were seeded and treated with various concentrations of propolin G (0, 5, 10, 15, and 20 μM) for 24 h. Thereafter, the cell viability was assayed by MTT methods. (**D**) MDA-MB-231 cells were treated with indicated concentrations of propolin G (0, 5, 10, 15, and 20 μM) for 24 h. Then, cell cycle distribution was determined by flow cytometer as described in Materials and Methods. Data were collected from at least triplicate experiments and the results represent the mean ± S.D. A significant difference is labeled as * when *p* < 0.05 between the control and the propolin G-added group.

According to Figure 1, MDA-MB-231 and Hs578T cells with high migration and invasion abilities were chosen to evaluate the anti-migration and anti-invasion effects of propolin G. The results reveal that the migration and invasion activities of MDA-MB-231 cells were suppressed by propolin G in a dose-dependent manner. However, the significant repression of migration and invasion activities of Hs578T cells was only detected in 10 μM of propolin G-treated cells (Figure 2A,B). The migration and invasion effects of propolin G was efficiently suppressed more in MDA-MB-231 than Hs578T cells.

To exclude the anti-migration and anti-invasion activities of propolin G resulting from the inhibition of cell viability, the cytotoxicity effect of propolin G was analyzed. Under 10 μM of propolin G treatment, the cell viability was not significantly affected in both MDA-MB-231 and Hs578T cells. However, decreasing cell viability was observed in more than 10 μM of propolin G treatment. Additionally, we also examined the cytotoxicity effect of propolin G in immortalized breast epithelial cells. Even with 20 μM of propolin G treatment, the cell viability of MCF10A was not significantly decreased (Figure 2C). Additionally, the cell cycle distribution was also inspected in propolin G-treated MDA-MB-231 cells. No significant difference in cell cycle accumulation was detected after 10 μM of propolin G treatment. However, G1 phase accumulation was perceived after 15 or 20 μM of propolin G treatment (Figure 2D). The results imply that the mechanisms of propolin G-suppressed migration and invasion of MDA-MB-231 cells were cytotoxicity independent.

### 2.3. Migration and Invasion Activities Suppressed by Propolin G in MDA-MB-231 Cells via Snail-Regulated EMT Signaling Pathway

**Figure 3:**
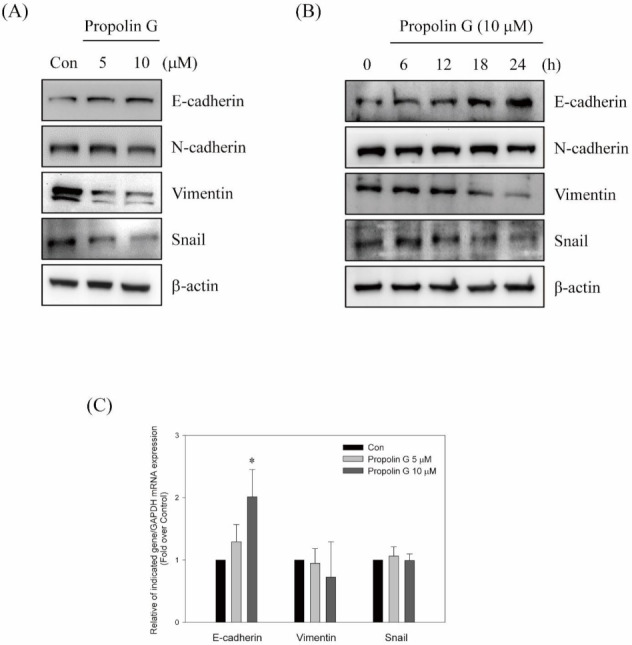

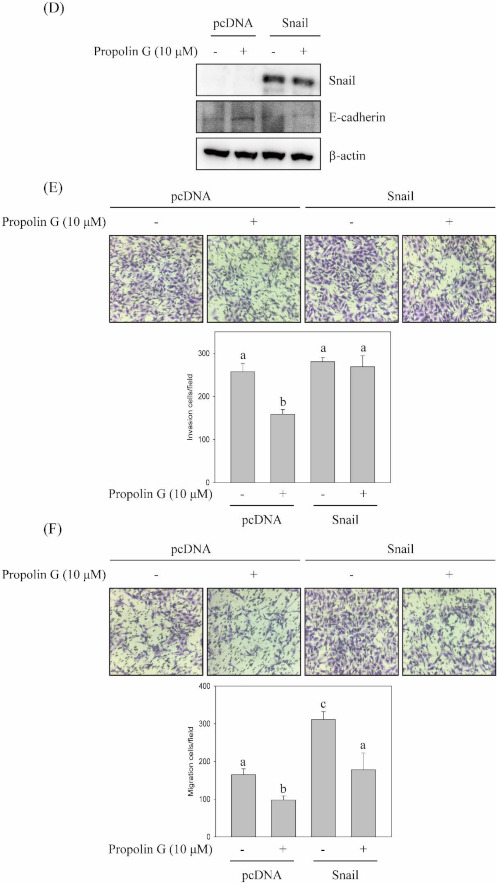
Snail-mediated migration and invasion activities inhibited by propolin G in MDA-MB-231 cells. MDA-MB-231 cell were incubated with (**A**) indicated concentrations of propolin G (0, 5, and 10 μM) for 24 h or (**B**) 10 μM of propolin G for different time interval. After treatment, the protein levels of indicated EMT molecules were analyzed by Western blot. (**C**) The mRNA expression of E-cadherin, vimentin, and Snail were inspected by quantitative real-time PCR. The relative indicated mRNA expressions were normalized with GAPDH as described in Materials and Methods. (**D**) MDA-MB-231 cells were briefly transfected with Snail plasmid. Subsequently, the cells were treated with or without propolin G for 24 h. The protein expression of Snail and E-cadherin were detected by Western blotting. (**E**) The migration and (**F**) the invasion activities were assayed by in vitro migration and invasion assay, respectively. Data were collected from at least triplicate experiments and the results represent the mean ± S.D. Different lowercase letters represent significant differences between the two groups when *p* < 0.05.

To understand the molecular mechanisms of anti-migration and anti-invasion by propolin G, the expression of EMT molecules was addressed. Since the inhibition of migration and invasion activities in MDA-MB-231 cells were more effective than in Hs578T cells with propolin G treatment (Figure 2), MDA-MB-231 cells were selected for further evaluation. As shown in Figure 3, the expression of E-cadherin was up-regulated by propolin G treatment in dose- and time-dependent patterns. Meanwhile, dose- and time-dependent inhibitions of vimentin and Snail expressions, rather than N-cadherin, were observed. (Figure 3A,B). In addition, the mRNA expression of E-cadherin was up-regulated after propolin G treatment. Nevertheless, the expressions of vimentin and Snail mRNA were not significantly different between control and propolin G-treated cells (Figure 3C). The results imply that the expressions of vimentin and Snail were inhibited by propolin G via post-translational regulation, rather than transcriptional regulation. The up-regulation of E-cadherin expression following the suppression of the Snail-dependent signaling pathway was speculated.

The Snail-regulated signaling pathway of propolin G-induced E-cadherin expression was further investigated. Ectopic expression of Snail was performed to address the issue. The outcomes show that propolin G-induced E-cadherin expression was suppressed by Snail overexpression (Figure 3D). Moreover, the role of Snail in propolin G-suppressed migration and invasion of MDA-MB-231 cells was assessed. As shown in Figure 3E,F, the repressions of MDA-MB-231 cell migration and invasion activities by propolin G were dramatically reversed by ectopic expression of Snail (Figure 3E,F).

### 2.4. Prompting Vimentin and Snail Degradation by Propolin G via Ubiquitin/Proteasome System

**Figure 4:**
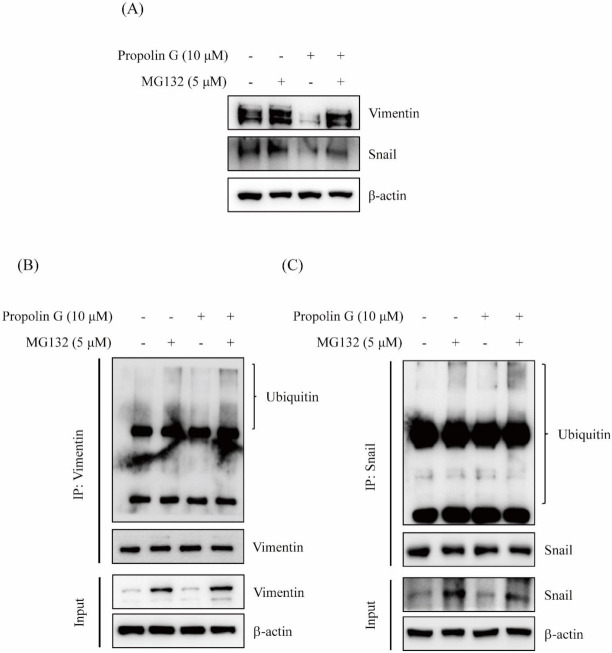
The expressions of Snail and vimentin inhibited by propolin G via protein degradation pathway in MDA-MB-231 cells. MDA-MB-231 cells were stimulated with propolin G for 24 h after proteasome inhibitor (MG132, 5 μM) pretreatment for 1 h. Then, collected cell lysates were subjected to analysis of (**A**) the expression of Snail and vimentin by Western blot. The cell lysates were immuno-precipitated by (**B**) vimentin or (**C**) Snail as described in Materials and Methods. Associated ubiquitin was detected by Western blot assay. Data represent at least three independent experiments.

Since the expressions of vimentin and Snail inhibited by propolin G were not regulated by the transcriptional mechanisms (Figure 3C), protein degradation was further investigated to understand the disruption of vimentin and Snail expression by propolin G. Cells were pretreated with proteasome inhibitor MG132 before propolin G incubation. Thereafter, the expressions of vimentin and Snail were detected by Western blotting. The results exhibit that propolin G-decreased vimentin and Snail expression was dramatically reversed by the pretreatment with MG132 (Figure 4A). The poly-ubiquitination statuses of vimentin and Snail in propolin G-treated cells by immuno-precipitation and Western blot analyses were also examined. The noticeable poly-ubiquitin accumulations of vimentin and Snail were perceived in propolin G and MG132 co-treatment (Figure 4B,C). The results show that the expressions of vimentin and Snail by propolin G treatment were reduced by disrupting protein stability following the ubiquitin–proteasome degradation.

### 2.5. Propolin G-Suppressed Snail-Mediated Migration and Invasion via GSK-3β Signaling Pathway

**Figure 5:**
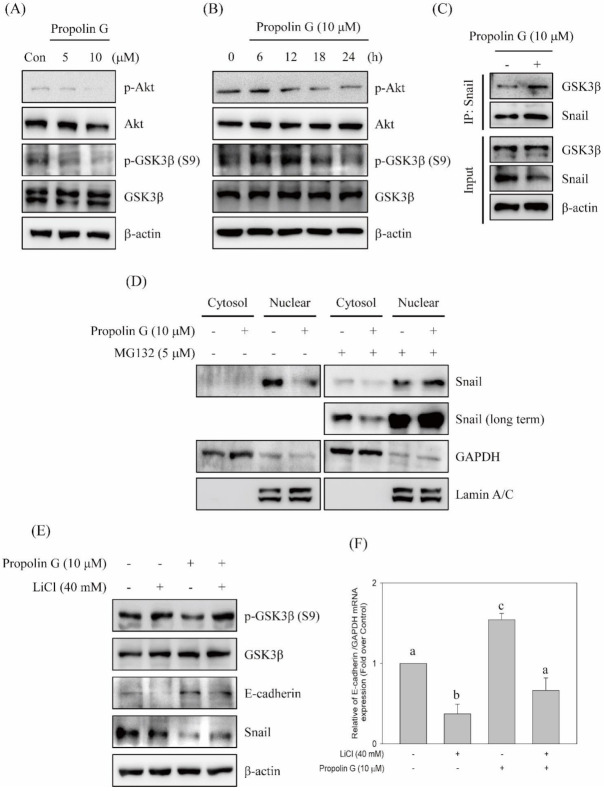

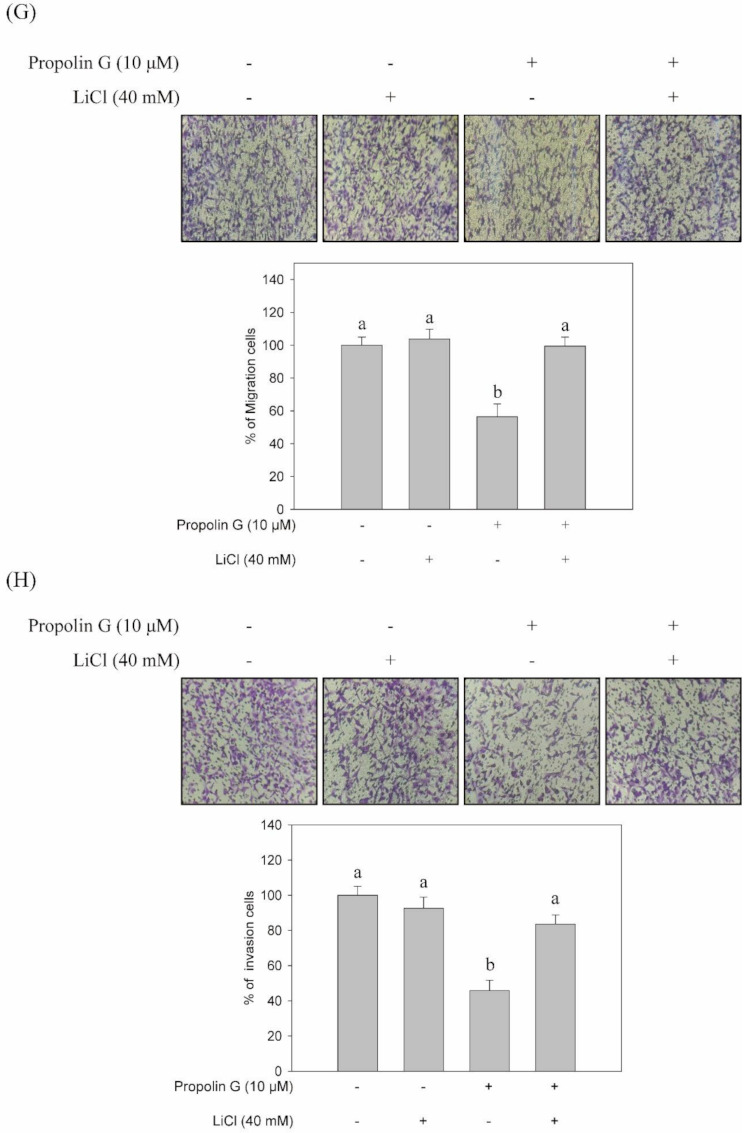
Effects of propolin G on GSK-3β-mediated Snail-dependent migration and invasion activities in MDA-MB-231 cells. MDA-MB-231 cells were incubated with (**A**) indicated concentrations of propolin G (0, 5, and 10 μM) for 24 h or (**B**) 10 μM of propolin G for different time intervals. The protein levels of p-Akt, Akt, p-GSK-3β, and GSK-3β were examined by Western blot. (**C**) The immuno-precipitation by Snail was conducted and the linked GSK-3β with Snail was evaluated by Western blot. (**D**) The cells were pretreated with or without MG132 (5 μM) for 1 h, and propolin G (10 μM) was added for 24 h subsequently. Snail, GAPDH, and Lamin A/C in the cell lysates of cytoplasm and nuclear fraction, respectively, were determined by Western blots. (**E**) MDA-MB-231 cells were stimulated with propolin G for 24 h after GSK-3β inhibitor (lithium chloride, LiCl) pretreatment for 1 h. The collected lysates were analyzed to inspect the indicated protein expressions and (**F**) the mRNA expression of E-cadherin. (**G**) The migration and (**H**) invasion activities of MDA-MB-231 cells were studied after lithium chloride and/or propolin G treatment as described in Materials and Methods. Data were collected from at least triplicate experiments, and the results represent the mean ± S.D. Different lowercase letters represent significant differences between the two groups when *p* < 0.05.

Post-translational modification by phosphorylation is a key mechanism to govern Snail protein stability, subcellular localization, and EMT process. Snail phosphorylation is shown in the GSK-3β-dependent mechanism [13,14]. The elevations of Snail instability and degradation were detected in propolin G-treated cells (Figure 4). To verify the role of protein phosphorylation modification in propolin G-promoted Snail degradation, the GSK-3β signaling pathway was investigated. The reduction in GSK-3β phosphorylation at Ser9 site negatively regulated GSK-3β activity and was dose- and time-dependently inhibited by the addition of propolin G (Figure 5A,B). The expression of phospho-Akt, a well-known kinase phosphorylates GSK3β at Ser9, was also inhibited by propolin G treatment (Figure 5A,B). Moreover, Snail was immuno-precipitated and followed by GSK-3β detection to reveal the signaling pathways in propolin G-induced Snail instability. The results show that the association of GSK-3β with Snail was dramatically increased in propolin G-treated cells (Figure 5C). In addition, the overexpression of Snail in nuclear fraction was found and suppressed by propolin G treatment. However, the propolin G-repressed Snail expression in nuclear fraction was recovered by proteasome activity inhibition (Figure 5D). Accordingly, propolin G promoted GSK-3β and Snail association directed to the disruption of Snail nuclear localization and then degradation.

To further explore the role of GSK-3β in propolin G-suppressed migration and invasion, the inhibition of GSK-3β activity by lithium chloride was performed. The results disclose that propolin G-inhibited Snail expression was rescued by GSK-3β activity inhibition. In the meantime, both the up-regulation protein and mRNA expression of E-cadherin in propolin G-treated cells were restrained by GSK-3β activity blockade (Figure 5E,F). Propolin G-suppressed cell migration, and invasion activities were evidently recovered after GSK-3β inhibitor pretreatment (Figure 5G,H).

### 2.6. Down-Regulated Expression of Vimentin by Propolin G through HDAC6-Mediated Signaling Pathway

**Figure 6:**
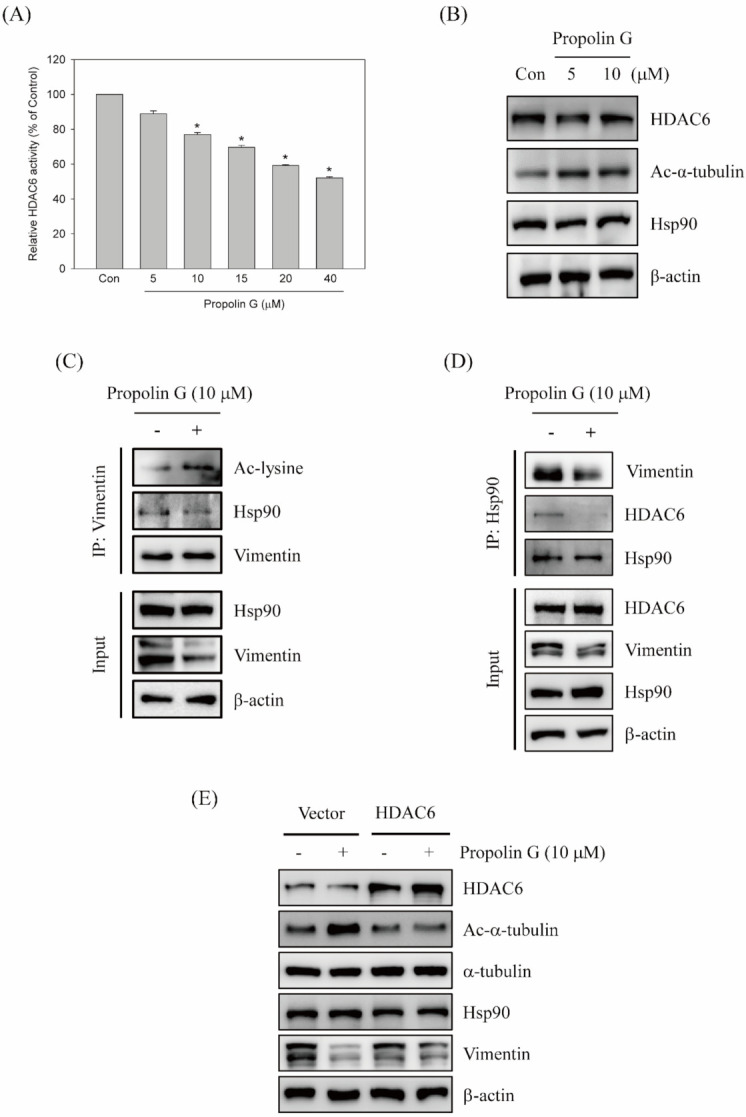

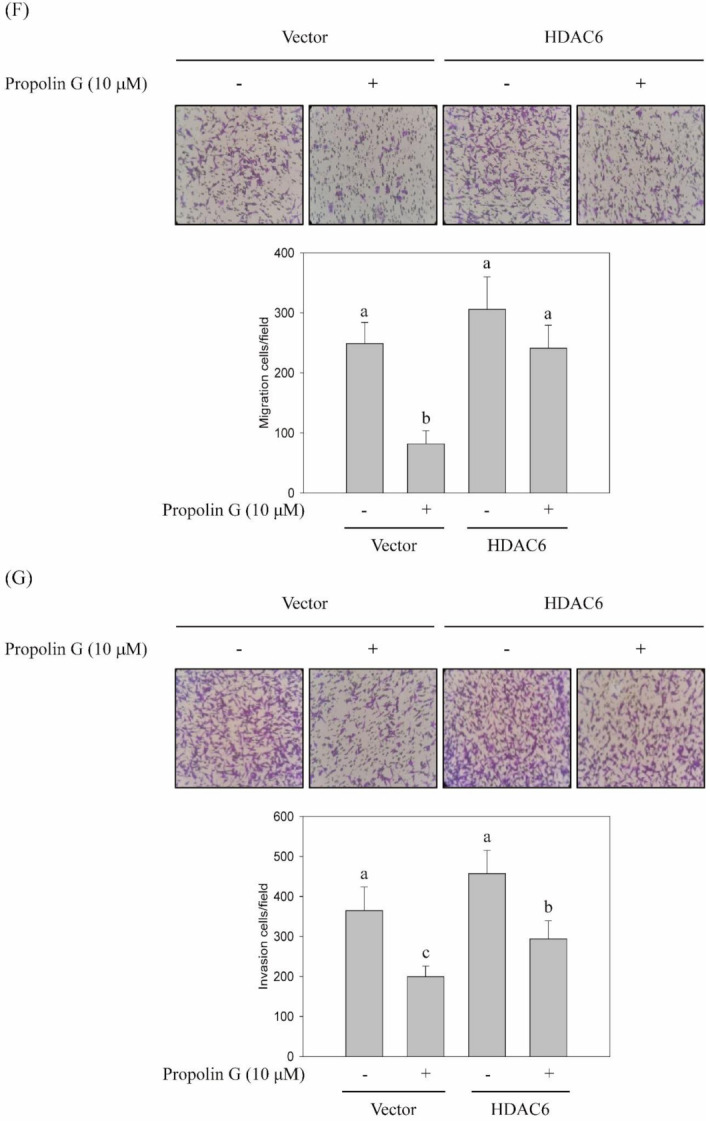
HDAC6-mediated migration and invasion restrained by propolin G in MDA-MB-231l cells. MDA-MB-231 cells were treated with indicated concentrations of propolin G (0, 5, 10, 15, 20, and 40 μM) for 24 h, and then cell lysates were harvested for (**A**) HDAC6 activity assay, and (**B**) the expressions of HDAC6, acetyl-α-tubulin, Hsp90, and β-actin were determined by HDAC6 activity assay kit and Western blot analyses, respectively. The cell lysates of the control and propolin G-treated cells were harvested. Acetyl-lysine, Hsp90, vimentin, and HDAC6 expressions were determined by Western blotting, while the lysates were immuno-precipitated by (**C**) vimentin or (**D**) Hsp90. (**E**) MDA-MB-231 cells were briefly transfected with HDAC6 plasmid. Afterward, the cells were treated with or without propolin G (10 μM) for 24 h. The expressions of HDAC6, acetyl-α-tubulin, Hsp90, and β-actin, in addition to (**F**) the migration and (**G**) the invasion activities, were examined by Western blot and in vitro migration and invasion protocols as described in Materials and Methods, respectively. Data were collected from at least triplicate experiments and the results are represented as the mean ± S.D. A significant difference is labeled as * when *p* < 0.05 between the control and the propolin G-added group. Different lowercase letters represented significant differences between the two groups when *p* < 0.05.

Protein modification by acetylation is another mechanism that affects protein stability. Recent studies indicate that invadopodia and invasion activities of breast cancer are controlled by HDAC6 [24]. Furthermore, vimentin filament collapse is regulated by HDAC6 linked to cell stiffness [25]. In addition, acetylation of vimentin is involved in hepatocellular carcinoma cell migration [26]. Since the down-regulation of vimentin expression with propolin G treatment was coordinated by a transcriptionally independent mechanism (Figure 3), the role of acetylation modification of vimentin expression in propolin G-treated cells was further measured. The effects on the HDAC6 signaling pathway by propolin G were initially examined. As shown in Figure 6A, HDAC6 activity in propolin G-treated MDA-MB-231 cells was suppressed in a dose-dependent manner. The expression of acetyl-α-tubulin, a downstream substrate of HDAC6, was up-regulated with propolin G incubation. However, the expression of HDAC6 was not affected by propolin G treatment (Figure 6B).

The acetylation of vimentin was explored by immuno-precipitation assay to assess the functions of HDAC6 activity inhibited by propolin G in vimentin stability. The results reveal that the acetyl-lysine of vimentin was apparently accumulated in propolin G-treated cells. Interestingly, the binding chaperone protein Hsp90 with vimentin was inhibited by propolin G supplement (Figure 6C). Moreover, Hsp90 was also immuno-precipitated, and then the interaction of vimentin expression was studied. The binding vimentin with Hsp90 repressed in the cells with the addition of propolin G is easily seen in Figure 6C (Figure 6D). Additionally, the association of HDAC6 and Hsp90 was dramatically repressed by propolin G, resulting in the destruction of HDAC6/Hsp90/vimentin complexes (Figure 6D). Convincingly, vimentin expression by propolin G was down-regulated via the inhibition of HDAC6 activity followed by the accumulation and degradation of acetylated vimentin.

### 2.7. Attenuation of Propolin G-Inhibited Migration and Invasion by the Ectopic Expression of HDAC6

Additionally, overexpression of HDAC6 was performed to address the HDAC6-mediated signaling pathway in propolin G-suppressed vimentin expression, cell migration, and invasion activities. The cells were transfected with HDAC6 plasmid or control vector before propolin G treatment. Afterward, the expressions of indicated proteins were detected by Western blotting. The results show that propolin G-induced acetyl-α-tubulin expression in MDA-MB-231 cells was reduced by HDAC6 overexpression. Meanwhile, the down-regulation of vimentin by propolin G was rescued by ectopic expression of HDAC6 (Figure 6E). The advanced verification of HDAC6 in propolin G-suppressed migration and invasion activities disclosed that repressed migration and invasion activities in MDA-MB-231 cells by propolin G were significantly recovered by HDAC6 overexpression (Figure 6F,G).

## 3. Discussion

Numerous studies indicate that the majority of cancer deaths are caused by metastasis. The proportion of deaths resulting from malignant metastasis is variable among primary cancer types. For example, about 10% of central nervous system cancers develop into fatality that is the result of cancer metastasis; however, testicular cancer metastasis almost causes 100% of deaths. Commonly, cancer metastasis causes about 67% of cancer deaths [42]. Although the enhancement in diagnosis and treatments of breast cancer demonstrate the reduction in deaths from metastasis, the overall survival rate of TNBC still shows insignificant improvement [43]. TNBC possesses high malignancy, relapse, and low survival rates among breast cancer subtypes [44]. Due to the lack of efficient target therapy, TNBC is now still a major health threat to the worldwide female population. Therefore, understanding the molecular mechanisms of cancer metastasis might provide the insight to improve survival from TNBC. The analyses of the correlations between the expression of EMT molecules and TNBC specimens show that the expressions of vimentin and transcription factors, such as Snail, Slug, and Twist, are significantly up-regulated, whereas the E-cadherin expression is repressed in TNBC tumors. Abnormal expressions of mesenchymal markers are found to be a greatly aggressive phenotype of TNBC. Furthermore, ectopic expressions of mesenchymal markers and low E-cadherin expression are highly connected to poor prognosis and poor survival rate of TNBC [45,46,47]. Accordingly, reversing or repressing EMT processes might be an appropriate approach for improving the prognosis and survival rate of TNBC. The migration and invasion activities as well as EMT marker expressions of TNBC cell lines were assessed to understand the relationship between EMT and aggressive properties of TNBC. The results show that MDA-MB-231 and Hs578T cells presented high migration and invasion activities. The expressions of mesenchymal markers of these two cell lines, N-cadherin, vimentin, and Snail were apparently discovered, while few expressions of E-cadherin were observed (Figure 1). Interestingly, this profile of EMT marker expressions conflicted with Snail expression in low aggressive TNBC cell lines. These aberrant expressions of mesenchymal markers might lead to highly aggressive properties of TNBC cells.

The activities of propolin G, a prenylflavanone component of Taiwanese propolis, on migration and invasion in MDA-MB-231 and Hs578T cells were inhibited in a cytotoxicity-independent measure (Figure 2). Moreover, the expressions of E-cadherin mRNA and protein were up-regulated in the cell treated with propolin G, although the protein expressions of vimentin and Snail were inhibited in dose- and time-dependent profiles. However, the mRNA expressions of vimentin and Snail were insignificantly discovered (Figure 3A–C). The consequences revealed that E-cadherin was increased by propolin G via transcriptional-dependent regulation, whereas vimentin and Snail were reduced via transcriptional-independent mechanisms.

Inhibition of E-cadherin expression by transcription factor Snail leading to increased cell mobility has been reported [48]. Ectopic expression of Snail has been discovered both in epithelial and endothelial cells of invasive breast cancer [49,50]. Furthermore, Snail expression is decidedly associated with tumor grade, nodal metastasis of invasive ductal carcinoma, and outcome prediction of breast cancer [48,51,52]. Blockade of Snail expression or activity is targeted to restrain EMT and cancer metastasis [53,54,55]. The role of Snail in propolin G-suppressed migration and invasion in MDA-MB-231 cells was appraised because propolin G-inhibited Snail expression had been observed in MDA-MB-231 cells through a dose- and time-dependent model (Figure 3 A,B). Propolin G-repressed migration and invasion activities in MDA-MB-231 cells were clearly rescued by transfection of ectopic expression of Snail (Figure 3D–F). The consequences exposed that migration and invasion were suppressed by propolin G via the Snail-dependent signaling pathway.

Post-translational modification by phosphorylation is the critical mechanism to regulate Snail activity [48,56]. Two phosphorylation motifs on exon 2 of the *snail* gene have been discovered [48]. The phosphorylation statuses of two motifs control Snail activity and stability. The motif 2 of Snail is bound and phosphorylated by GSK-3β and then by the forced nuclear exportation of Snail. Subsequently, GSK-3β continuously phosphorylates the residue at motif 1 on Snail and then is associated with β-Trcp followed by Snail degradation [48]. Many oncogenic kinases, such as Akt, MAPK, and Wnt, stabilize Snail protein expression, nuclear localization, and triggering of EMT. Consequently, restrained GSK-3β activation results in cell migration and invasion [13,48,56,57,58]. Thus, activation of the pivotal target, GSK-3β activity, subsequently disrupted Snail expression and Snail-regulated EMT. Proteasome inhibitor treatment was conducted to verify that propolin G-suppressed Snail-mediated malignancy through the disruption of protein stability. As shown in Figure 4, Snail expression reduced by propolin G was recovered by proteasome inhibitor treatment. Meanwhile, Snail bound with ubiquitin was evidently exposed in propolin G and MG132 co-treatment (Figure 4). Furthermore, the expression of p-GSK-3β was inhibited by propolin G. Interestingly, the complex of Snail and GSK-3β was increased in propolin G-treated cells (Figure 5C). The cellular localization demonstrated that the expression of Snail in nuclear fraction was dramatically diminished by propolin G treatment. However, inhibition of Snail expression by propolin G in nuclear fraction was fully recovered by suppressing the proteasome degradation system (Figure 5D). The pivotal role of the GSK-3β-dependent pathway in propolin G-suppressed migration and invasion in MDA-MB-231 cells was also confirmed. Meanwhile, p-GSK-3β and Snail expression repressed by propolin G was rescued by lithium chloride, an inhibitor of GSK-3β, pretreatment. Surprisingly, the expressions of E-cadherin protein and mRNA were significantly recovered in the lithium chloride and propolin G co-treatment group (Figure 5E,F). In the meantime, propolin G-repressed migration and invasion activities were fully rescued by lithium chloride addition (Figure 5G,H). The outcomes strongly imply that Snail-regulated EMT, migration, and invasion activities by propolin G were inhibited via GSK-3β-directed Snail association, followed by Snail phosphorylation resulting in protein instability ensuring the degradation by ubiquitin–proteasome system. Additionally, it has been reported that the phosphorylation of vimentin on Ser39 governs cancer metastasis via Akt-dependent signaling pathway [17]. As shown in Figure 5A,B, the expression of p-Akt was dose- and time-dependently blocked by propolin G incubation. Therefore, the disruption of vimentin by phosphorylation in propolin G-suppressed migration and invasion activities of TNBC through the Akt-dependent pathway could not be excluded in the present study. Further examination should be carried out to verify this issue.

Epigenetic acetylation modification of histone and non-histone proteins on lysine residues is an important mechanism to regulate tumorigenesis and metastasis. Histone acetyltransferases (HATs) and histone deacetylases (HDACs) are two major enzymes overseeing acetylation and deacetylation at lysine residues on the target protein, respectively [59]. Many studies show that over-expression of HDAC isozymes in cancer cells and the ectopic expressions are correlated with poor prognosis and high malignancy [60,61,62,63]. HDAC6, a member of class IIb HDAC, has been disclosed as an interesting target for cancer therapy [64]. Overexpressed HDAC6 has been connected with cell migration and invasion in breast cancer, especially in TNBC [65,66]. HDAC6-regulated breast cancer cell invadopodia formation and invasion activity are also perceived [24]. Interestingly, acetylation status of vimentin regulates the migration and invasion activities in cancer cells [25,26]. It is postulated that acetylation modification mediated by HDAC6 might participate in the regulation of vimentin stability linked with propolin G since the expression of vimentin was inhibited by propolin G via post-translation-dependent regulation (Figure 3 and Figure 4). Additional investigations revealed that HDAC6 activity was dose-dependently suppressed by propolin G and that HDAC6 protein down-regulation was excluded (Figure 6A,B). Moreover, the complex of vimentin and Hsp90 was decreased, and the expression of vimentin acetylation was clearly detected after propolin G treatment by Western blotting analyses following immune-precipitation (Figure 6C,D). Moreover, the exploration of the association of HDAC6/Hsp90 with vimentin showed that the interaction of Hsp90 with HDAC6 was dramatically diminished by propolin G incubation (Figure 6D). The ectopic expression of HDAC6 was implemented to verify the role of HDAC6 in propolin G-suppressed migration and invasion (Figure 6E). The outcome unveiled that the repression of migration and invasion activities by propolin G was significantly recovered by HDAC6 overexpression (Figure 6F,G). Accordingly, inhibition of HDAC6 activity by propolin G led to hyper-acetylation of vimentin followed by disruption of vimentin resulting in instability, degradation, suppression of cell migration, and invasion thereafter.

**Figure 7:**
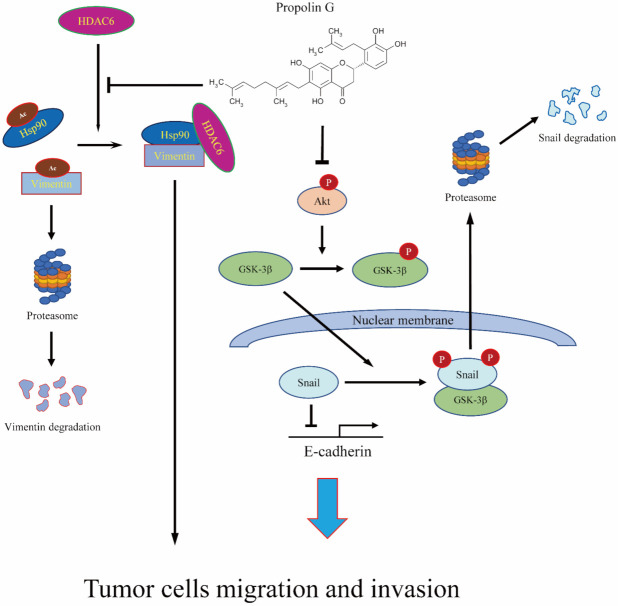
The schematic of the molecular mechanisms of anti-migration and anti-invasion activities of propolin G in TNBC cells.

Propolis, a safe and popular material, has been used as a folk medicine for many years. Numerous studies have shown that propolis retains brilliant anti-cancer properties. Propolis is suggested as a good chemopreventive agent. The assorted active compounds in propolis come from diverse plant exudates by bees’ harvest [27]. The c-prenylflavanones include eight members. Among them, one of them called propolins (or nymphaeols), has been identified from Pacific propolis, such as Taiwanese and Okinawan propolis [31,32,67,68]. Propolin G has been designated as having anti-cancer activity through apoptotic induction and liver protection ability by attenuating the TGF-β-regulated Smad2/3 signaling pathway [39,69]. Although propolin C, a biologically active analog of propolin G from Taiwanese propolis, shows activity that represses EMT-regulated migration and invasion via epidermal growth factor receptor (EGFR)-dependent pathway [41], the repression effects of propolin G on EMT-regulated cancer malignancy is still unclear, especially in breast cancer. The present study reports that the EMT-mediated migration and invasion activities of TNBC were blocked by propolin G. Down-regulation of Snail and vimentin protein expressions by propolin G was through post-translational modification mechanisms. It was unprecedented to demonstrate that GSK-3β and HDAC6 might be the molecular targets of propolin G. The migration and invasion activities of MDA-MB-231 cells were inhibited by activating GSK-3β-suppressed Snail and by disrupting HDAC6-regulated vimentin stability (Figure 7). The outcomes provide the possible mechanism of propolin G on anti-migration and invasion activities of TNBC cells. Propolin G could be considered a candidate chemopreventive or therapeutic agent for TNBC therapy.

## 4. Materials and Methods

### 4.1. Chemicals, Reagents, and Antibodies

Anti-HDAC6, anti-acetyl-α-tubulin, anti-acetyllysine, anti-Snail, and anti-GSK-3β antibodies were obtained from Cell Signaling Technology (Beverly, MA, USA). Mouse anti-β-actin, anti-Hsp90, anti-ubiquitin, and protein A/G plus agarose were gained from Santa Cruz Biotechnology (Santa Cruz, CA, USA). Anti-N-cadherin was purchased from Thermo Fisher Scientific Inc., (Taipei, Taiwan). Anti-vimentin and anti-E-cadherin antibodies were acquired from GeneTex, Inc. (Irvine, CA, USA) and BD Biosciences, Inc. (San Jose, CA, USA), respectively.

### 4.2. Purification of Propolin G

The purification of propolin G was modified from Pai. et al. [41]. Briefly, Taiwanese propolis (985 g, Hualian, Taiwan) was extracted with 5 L of ethanol six times at room temperature. The collected ethanol extracts were filtered and concentrated to obtain a brown dense syrup (902.2 g). The dense ethanol extract was eluted by gradient buffer (CH_2_Cl_2_ and EtOAc) on a column (column: 14 cm i.d. × 75 cm) packed with silica gel (SiliaFlash G60, Silicycle). Seven fractions were collected. Thereafter, fraction 2 was then applied on a silica gel column and eluted with a gradient of n-hexane and acetone to obtain propolin G (698.5 mg). The structure of propolin G was identified by the spectral data in the literature [39]. All propolins concentrations of the dense ethanol extract were listed (Supplementary Table S1).

### 4.3. Cell Culture and Cell Viability Assays

TNBC cell lines MDA-MB-231, MDA-MB-468, Hs578T, HCC1937, and non-tumorigenic mammary epithelial cells, MCF10A, were kindly provided by Prof. Yuan-Soon Ho (Taipei Medical University). All of TNBC cell lines were maintained in DMEM-F12 (Hyclone Laboratories, Logan, UT, USA) containing 10% fetal bovine serum and 5% CO_2_ atmosphere at 37 °C. MDA-MB-231, Hs578T, and MCF10A cells (1 × 10^4^/well) were seeded in 96-well plates and then treated with different concentrations of propolin G (5, 10, 15, and 20 μM) for 24 h. Afterward, MTT assay was performed to evaluate cell viability.

### 4.4. Cell Cycle Analyses

MDA-MB-231 cells (2 × 10^5^ cells/well) were cultured and treated with different concentrations of propolin G (5, 10, 15, and 20 μM) for 24 h. Subsequently, cells were collected, washed, and stained with propidium iodide (50 μg/mL, Sigma Chemical, St. Louis, MO, USA). The DNA content was detected by FACScan laser flow cytometer analysis system, and the cell cycle distribution was analyzed by Kaluza analysis software (Beckman Coulter, Fullerton, CA, USA).

### 4.5. In Vitro Invasion and Migration Assays

In vitro migration and invasion assays were analyzed by modified protocols from Pai et al. [41]. Briefly, TNBC cells were treated with or without indicated concentrations of propolin G (5 and 10 μM) for 24 h. Cells were then harvested to seed on a Boyden chamber (BD Bioscience, Bedford, MA, USA) at a cell density of 1 × 10^5^ cells/well following the incubation in serum-free medium for 24 h. For migration assay, the lower chamber of polycarbonate filters (8 μm pore) were filled with 5% FBS containing RPMI-1640 medium. After 24 h incubation, the cells that migrated to the lower chamber were washed, fixed with methanol, and stained with 0.1% crystal violet, subsequently. The photography of the migrated cells was taken under a light microscope. Subsequently, the cell numbers were counted for statistical analyses. An in vitro invasion assay was carried out as the described in vitro migration assay with coating Matrigel (25 mg/mL) on polycarbonate filters.

### 4.6. Transfection

The manufacture’s instruction for transfection was followed. Briefly, MDA-MB-231 cells were seeded and grown to about 70% confluence and then the empty vector, HDAC6 plasmid (constructed by Prof. Kuo-Tai Hua, National Taiwan University), or Snail plasmid (a gift from Prof. Ming-Hsien Chien, Taipei Medial University) was transfected by Lipofectamine 2000 Transfection Reagent (Thermo Fisher Scientific Inc., Taipei, Taiwan). After transfection, cells were treated with or without the indicated concentration of propolin G. The cells were ready for the following experiments.

### 4.7. Quantitative Real-Time Reverse Transcription Polymerase Chain Reaction (qRT-PCR)

After treatment with propolin G, total RNA was collected by the RNA mini kit (Qiagen, Taipei, Taiwan), and the cDNAs were synthetized by commercial cDNA reverse transcription kit (Invitrogen, Taipei, Taiwan). qRT-PCR was conducted by PrecisionPLUS qPCR Master Mix premixed with SYBRgreen (Primerdesign, Camberley, UK) on MyGo Pro RT-PCR system (IT-IS Life Science, Dublin, Ireland). The primer sequences of real-time PCR are shown below: E-cadherin Forward: 5′-TCGGCCTGAAGTGACTCGTA-3′, Reverse: 5′-CGGAACCGCTTCCTTCATAG -3′; vimentin Forward: 5′-TGCCCTTAAAGGAACCAATG -3′, Reverse: 5′-TCCAGCAGCTTCCTGTAGGT-3′; Snail Forward: 5′-CGGAAGCCTAACTACAGCGA-3′, Reverse: 5′-GCCAGGACAGAGTCCCAGAT-3′; GAPDH Forward: 5′-CTCTGGTAAAGTGGATATTGT-3′, Reverse: 5′-GGTGGAATCATATTGGAACA-3′. The relative mRNA expressions were calculated by the 2^−ΔΔCt^ value and normalized to the internal control of GAPDH.

### 4.8. Immuno-Precipitation

The immuno-precipitation protocols were modified from Liao et al. [70]. One milligram of total proteins was interacted with protein-A/G agarose bead for preclear at 4 °C for 30 min. After the first preclear the non-specific binding, the supernatant was collected, and the indicated antibody was added for reaction at 4 °C for 2 h. Thereafter, immuno-complexes were incubated with rabbit anti-mouse IgG for the secondary preclear process at 4 °C for 30 min. The mixtures were harvested and then reacted with protein-A/G agarose bead overnight. After that, the immuno-complexes were washed three times with immunoprecipitation buffer and then extracted with RIPA extraction buffer. Western blot analyses were implemented to detect the indicated protein expressions.

### 4.9. In Vitro HDAC Activity Detection

In vitro HDAC6 activity was detected by the Fluor-de-Lys^®^ HDAC6 fluorometric drug discovery kit (Enzo Life Sciences, Farmingdale, NY, USA), and the manufacturer’s protocol was followed to analyze the data. Briefly, propolin G-treated MDA-MB-231 cell lysates were incubated with HDAC6 assay substrates at 37 °C for 15 min. Afterward, the reaction was stopped with HDAC6 developer buffer at 37 °C for 45 min, and the fluorescence was measured by TECAN microplate readers (Tecan Trading AG, Männedorf, Switzerland). The emission wavelength at 460 nm and excitation wavelength at 360 nm were selected for detection.

### 4.10. Western Blot Analyses

After the cells incubated with or without indicated concentrations of propolin G, the cells were harvested, and proteins were extracted by RIPA buffer. The total proteins were quantitated by Bradford reagent (Bio-Rad, Hercules, CA, USA). Equal amounts of proteins were separated by sodium dodecyl sulfate-polyacrylamide gel electrophoresis (SDS-PAGE). After separation, proteins were transferred to polyvinylidene difluoride membranes (Millipore, Billerica, MA, USA), and the membranes were then blocked and incubated with the primary antibodies at room temperature for 2 h. After incubation, membranes were washed, and secondary antibodies were subjected to further reaction. ECL reagent was used to detect the chemiluminescence signals (Amersham Pharmacia Biotech, Chicago, IL, USA), and thereafter the image was investigated by UVP ChemiDoc-It2 810 Imager (UVP, Upland, CA, USA) system.

### 4.11. Statistical Analyses

The results are represented as the mean ± SD from at least three independent experiments. The significant differences were analyzed by one-way ANOVA and followed by post hoc tests. Statistical significant was determined by *p*-value < 0.05.

## 5. Conclusions

Propolin G-inhibited migration and invasion activities in TNBC cells were through activation of GSK-3b-mediated Snail degradation. Meanwhile, vimentin stability regulated by HDAC6 was also disrupted by propolin G. Therefore, it is suggested that propolin G might serve as a therapeutic and/or chemopreventive agent for TNBC treatment.

## Data Availability

The data presented in this study are available in this article.

## Supplementary Materials

The following are available online at www.mdpi.com/ijms1568300/s1.
