# Supplementary material for: Propolin G-Suppressed Epithelial-to-Mesenchymal Transition in Triple-Negative Breast Cancer Cells via Glycogen Synthase Kinase 3β-Mediated Snail and HDAC6-Regulated Vimentin Degradation
Source: Int J Mol Sci. 2022 Jan 31;23(3):1672. doi: 10.3390/ijms23031672 (PMC8835855; doi:10.3390/ijms23031672)
Supplement: Supplementary file 1 [file ijms-23-01672-s001.zip › ijms-1568300-supplementary.pdf]

Supplementary Table S1 Chemical composition of Taiwanese propolis extract.

| Propolins | Weight (mg) |
|---|---|
| Propolin A | 3052.2 |
| Propolin C | 20106.4 |
| Propolin D | 5558.1 |
| Propolin E | 4.9 |
| Propolin F | 71.7 |
| Propolin G | 698.5 |
| Propolin H | 1470.1 |
| Prokinawan | 4.6 |
